# Humoral Immunity to Measles, Mumps, Rubella, Diphtheria, Tetanus and Pertussis After Cancer Treatment in Children

**DOI:** 10.1002/cnr2.70155

**Published:** 2025-05-19

**Authors:** Susanna Sundell, Miia Laine, Liisa Järvelä, Ville Peltola, Tytti Vuorinen, Päivi Lähteenmäki, Linnea Schuez‐Havupalo

**Affiliations:** ^1^ Department of Pediatrics and Adolescent Medicine Turku University Hospital and University of Turku Turku Finland; ^2^ Department of Clinical Microbiology Turku University Hospital and University of Turku Turku Finland; ^3^ Institute of Biomedicine, University of Turku Turku Finland

**Keywords:** child, cytostatic agents, hematologic neoplasms, immune reconstitution, neoplasms, vaccination

## Abstract

**Background:**

Treatment for pediatric malignancies has distinct effects on the immune system.

**Aims:**

Our aim was to measure humoral immunity to measles, mumps and rubella (MMR), and diphtheria, tetanus and acellular pertussis (DTaP) vaccines after pediatric cancer treatment.

**Method:**

We assessed IgG titers of 52 children against the vaccine antigens post‐treatment.

**Results:**

After completed primary vaccination series for MMR and DTaP before treatment, post‐treatment there was a considerable proportion of patients with below‐reference titers against pertussis, diphtheria and tetanus, but only few subjects showed below‐reference titers against measles, mumps and rubella. Our findings may have implications when considering re‐vaccination policies.

**Abbreviations**
CD: Cluster of differentiation
DTaP: Diphtheria, tetanus, acellular pertussis
EIA: Enzyme immunoassay
Hib: Hemophilus influenzae B
Ig: Immunoglobulin
IPV: Inactivated polio vaccine
ITR‐3: Intensity of Treatment Rating Scale
MMR: Measles, mumps, rubella
SD: Standard deviation

## 1 Introduction

Treatment of pediatric malignancies has advanced within the last decades. With increasing cure rates, reducing treatment‐related toxicity has become an important aim. One of the major challenges of cancer treatments is their effect on the immune system [1]. Loss of vaccine‐induced protection against infectious diseases after cancer therapy constitutes a potential long‐term sequela of treatment in an already vulnerable population. Re‐vaccination has been shown to be effective [2, 3, 4, 5, 6]. To prevent disease, evidence‐based plans for re‐vaccination should be available for each patient [1, 6, 7].

While there are national re‐vaccination schedules after stem cell transplantations, the evidence behind re‐vaccination policies after non‐transplant chemotherapy is much sparser [1]. Practices are diverse [8, 9], although some ground rules have been established [1, 10, 11, 12, 13, 14]. Many centers apply routine booster vaccines around 6 months after therapy and at a later time point for live vaccines [1]. For the live measles, mumps and rubella (MMR) vaccine, a time interval of 6–24 months post‐treatment is applied by many institutions [10, 15].

Our aim was to explore antibody titers post‐treatment in pediatric subjects having been previously immunized with the diphtheria, tetanus and acellular pertussis (DTaP), and MMR vaccines.

## 2 Methods

### 2.1 Study Population

This work was conducted in the form of a quality evaluation procedure as a retrospective cohort study. During the time frame of 2014–2019, there were 119 children starting chemo‐ and/ or radiotherapy treatment for malignant diseases at the Turku University Hospital and completing their treatment before April 2022. The study cohort has been described earlier [16]. Of the 79 children included in the study, there were 52 children with known data on a completed vaccination schedule for their age, who had not received immunoglobulin treatment within 6 months prior to analysis of immunoglobulin titers. Those 52 children made up the cohort of our analyses with available numbers for specific vaccine responses given in Tables 1 and 2. All patients were between the ages of 1–16 years at diagnosis, and all were treated either by chemotherapy alone or in combination with radiotherapy.

**TABLE 1:** Status of tetanus‐, *bordetella pertussis* ‐ and diphtheria antibodies according to treatment groups.

	*n*/*N* (%)	Low intensity treatment group *n*/*N* (%)	High intensity treatment group *n*/*N* (%)	*p*
Below‐reference tetanus antibodies	13/ 48 (27)	10/ 31 (32)	3/17 (18)	0.33**
Below‐reference pertussis antibodies	31/ 36 (86)	20/ 24 (83)	11/ 12 (92)	0.50*
Below‐reference diphtheria antibodies	17/ 34 (50)	12/ 22 (55)	5/12 (42)	0.72**
Time since previous PDT vaccine in years, mean (SD)	48 children	2.39 (2.77)	2.82 (3.0)	0.61***
Below‐reference total IgG by 4 months post‐treatment	6/48 (13)	3/ 32 (9)	3/ 16 (19)	0.39**
Below‐reference CD19 cell count by 4 months post‐treatment	2/49 (4)	2/32 (6)	0/17	NA

*Note:* Antibody status was assessed at 3–7 months post‐treatment. Included are children of any age who received at least 3 DTaP vaccines. Below‐reference CD19, CD19 count below 90 E6/l by 4 months post‐treatment. Low and high intensity treatment groups as defined earlier [16].

* Chi‐Square.

** Fisher's exact test.

*** *t*‐Test.

**TABLE 2:** Status of mumps, measles and rubella antibodies according to treatment groups.

	*n*/*N* (%)	Low intensity treatment group	High intensity treatment group	*p*
1–6‐year‐old children				
Below‐reference mumps antibodies	4/25 (16)	2/19 (11)	2/6 (33)	0.23 *
Below‐reference measles antibodies	2/ 26 (8)	2/20 (10)	0/ 6	NA
Below‐reference rubella antibodies	7/25 (28)	5/19 (26)	2/6 (33)	1.0 *
Time since previous MMR vaccine in years, mean (SD)	26 children	2.30 (1.59)	1.83 (1.72)	0.54 **
Below‐reference total IgG by 4 months post‐treatment	2/28 (7)	0/21	2/7 (29)	0.06 *
Below‐reference CD19 cell count by 4 months post‐treatment	0/28	0/21	0/7	NA
Whole cohort				
Below‐reference mumps antibodies	1/18 (6)	0/9	1/9 (11)	NA
Below‐reference measles antibodies	1/19 (5)	0/ 10	1/9 (11)	NA
Below‐reference rubella antibodies	1/18 (6)	1/9 (11)	0/9	NA
Time since previous MMR vaccine in years, mean (SD)	19 children	5.90 (3.54)	4.0 (3.35)	0.25 **
Below‐reference total IgG by 4 months post‐treatment	4/19 (21)	3/10 (30)	1/9 (11)	0.58*
Below‐reference CD19 cell count by 4 months post‐treatment	2/20 (10)	2/10 (20)	0/10	NA

*Note:* Antibody status was assessed at 3–7 months post‐treatment. Below‐reference CD19, CD19 count below 90 E6/l by 4 months post‐treatment. Rows 1–6: 1–6 years old children who had received at least 1 MMR vaccine. Rows 7–12: Children who had received 2 MMR vaccines, no age limit. NA, not applicable. Low and high intensity treatment groups as defined earlier [16].

* Fisher's exact test.

** *t*‐Test.

### 2.2 Study Conduct

We collected data on patient demographics, treatment details, intensity of treatment rating scale (ITR‐3) [16, 17], and immunological parameters. IgG antibody levels against measles, mumps and rubella viruses, *Bordetella pertussis* , *Corynebacterium diphtheriae* , and *Clostridium tetani* were measured 3–7 months post‐treatment, with most measurements performed 3–6 months post‐treatment.

The Finnish national vaccination program is voluntary and free of charge to everyone. Vaccine uptake in Finland is high with over 95% for the first MMR and 97.5% for the first DTaP vaccine in Southwest Finland (calculated for children born 2015) [18]. The schedule for DTaP and MMR is shown in Table S1 [18, 19]. Children who had received 3 or more doses of DTaP containing vaccine and 2 doses of MMR vaccine before cancer treatment were defined as fully vaccinated.

### 2.3 Quantification of Specific Antibody Levels

Measles and mumps virus IgG antibodies were analyzed with laboratory‐developed enzyme immunoassays (EIA) according to standard protocols using the BEP III analyzer (Siemens Healthcare, Germany) [20, 21, 22]. Rubella virus IgG antibodies were analyzed with a commercial enzyme linked fluorescent assay (Vidas, BioMerieux, France) according to the manufacturer's instructions. *Bordetella pertussis, Corynebacterium diphtheriae*, and *Clostridium tetani* IgG antibodies were analyzed with laboratory‐developed enzyme immunoassays using *Bordetella pertussis* whole‐cell sonicate, and diphtheria and tetanus toxoid antigens, respectively, with the Thermo Multiskan GO Spectrophotometer (Thermo Fisher Scientific, USA).

The following cut‐off values were used for a positive result: measles virus and mumps virus, IgG titer ≥ 40 (dilution 1:40); rubella virus, ≥ 15 IU/mL; *Bordetella pertussis and* *Corynebacterium diphtheriae* , absorbance ≥ 0.400 (semiquantitative result, +); *Clostridium tetani*, ≥ 0.1 IU/mL. Results at the cut‐off level were defined as positive.

Measles and mumps EIA cut‐off values in the laboratory developed methods were calculated as follows. Serum samples obtained from infants at the age of 6–7 months, when maternal antibody levels in sera of infants are low, were used as negative control samples. Positive control samples were from children and adults who had been vaccinated against measles and mumps. The samples were originally tested in the laboratory for other diagnostic purposes than measles or mumps infection. Serum samples from patients having measles or mumps were also used as positive controls in the development of EIA tests. The optical densities of negative control sera were determined in four times serial dilutions from 1:40 onward. The mean and standard deviation of negative sera were calculated and the cut‐off value was taken as three times the standard deviation. The optical density and the corresponding serum dilution above the cut‐off value was considered positive. For measles, mumps, and rubella viruses, the analyses were performed in the virus diagnostic laboratory at the Department of Virology, University of Turku, Finland. For *Bordetella pertussis* , *Corynebacterium diphtheriae* , and *Clostridium tetani*, the analyses were performed at the Department of Medical Microbiology and Immunology, University of Turku, Finland. From 2015, all analyses were carried out at Turku University Central Hospital Laboratories, Turku, Finland.

Laboratory methods to determine the absolute number of B cells (CD19+) have been described earlier [16]. Total IgG in plasma was quantified by immunoturbidimetry (Cobas 8000, Roche Diagnostics).

### 2.4 Statistics

In our analyses *p*‐values less than 0.05 were regarded as statistically significant.

Post‐treatment specific antibody titers within the reference range versus specific antibody titers below the reference range were compared stratifying by treatment intensity groups using the Chi‐Square test (Fisher's exact test for small numbers). Cases with below‐reference total IgG levels post‐treatment, those with low B‐cell numbers, and time since previous vaccination were compared between treatment intensity groups, the latter using the t‐test. In a further step, the effect of treatment intensity, time since previous vaccination, and below‐reference total IgG levels on specific post‐treatment antibody levels against pertussis, diphtheria and tetanus were estimated with adjusted logistic regression analyses. The variable of post‐treatment low B‐cell numbers had to be omitted due to zero cases in some categories. Adjusted odds ratios (aOR) with 95% confidence intervals (CI) were determined. Adjusted logistic regression was also performed to estimate effects of treatment intensity and time since previous vaccination on specific post‐treatment antibody levels against mumps and rubella. The variable of post‐treatment low B‐cell numbers and total IgG had to be omitted for the above‐mentioned reason. Due to zero cases in some categories, adjusted logistic regression analyses could not be performed to estimate effects on post‐treatment specific antibodies for measles.

All statistical analyses were performed using IBM SPSS Statistics for Windows, version 27 (IBM Corp., Armonk, NY).

### 2.5 Ethics

All the data analyzed were collected as part of routine diagnostics and treatment. Data analysis was carried out in connection with a quality evaluation procedure. Permission for the quality evaluation and to use hospital data was granted from the review board of the hospital district of Southwest Finland, permit number TO8/027/19. Data were handled in a strictly anonymous manner.

## 3 Results

**FIGURE 1:**
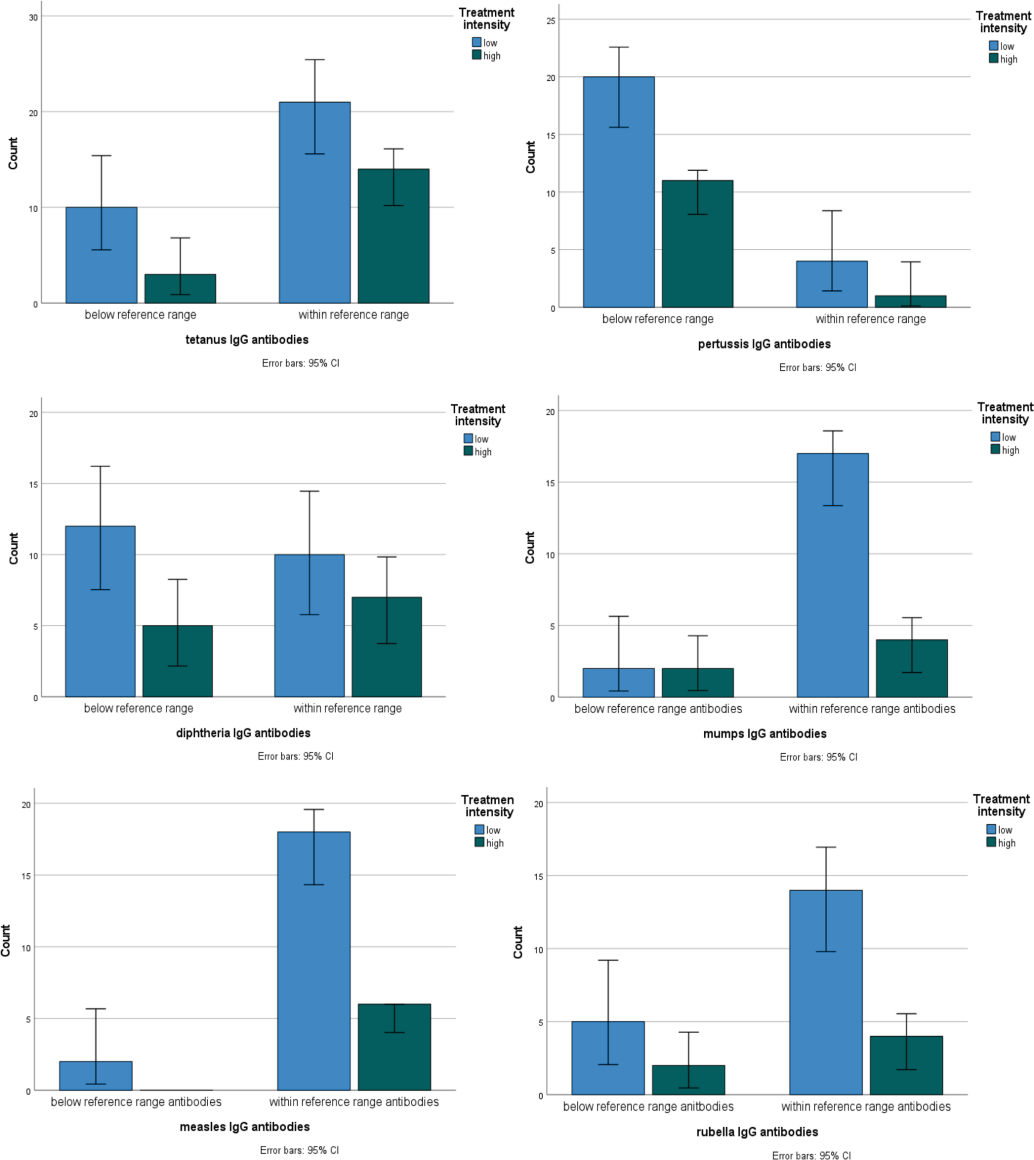
Bar‐diagrams showing distribution of patients (*N*) with respect to specific antibodies for tetanus, pertussis, diphtheria, mumps, measles, and rubella according to treatment intensity. Antibody status was assessed at 3–7 months post treatment. Mumps, measles, and rubella shown for children 1–6 years of age.

Of children fully vaccinated pre‐treatment (i.e., 3 DTaP vaccines and 2 MMR vaccines), 27% showed below‐reference antibody levels against tetanus, 86% against pertussis, 50% against diphtheria, 6% against mumps, 6% against rubella, and 5% against measles after cancer chemotherapy. Proportions of children with below‐reference total and specific IgG antibody levels post‐treatment, B‐cell (CD19+) recovery post‐treatment, and time since previous vaccination in groups divided by intensity of treatment are shown in Tables 1 and 2. Below‐reference titers were more common post‐treatment for DTaP than for MMR (Figure 1). B‐cells (CD19+) had recovered for the majority of patients. There were no significant differences between vaccination titers when compared between high and low intensity treatment groups. Adjusted logistic regression did not show any confounding effects between variables on post‐treatment specific pertussis, diphtheria, tetanus, mumps and rubella antibody levels (Table S2). Time from previous vaccination was not related to specific antibody status (Table S3).

## 4 Discussion

After completed cancer chemotherapy in children fully vaccinated for MMR and DTaP, there was a considerable proportion of children with below‐reference titers against pertussis, diphtheria and tetanus, but only few subjects showed below‐reference titers against measles, mumps and rubella. While a rapid decline in titer against pertussis antibodies is known also in the healthy pediatric population [23, 24], previous research indicates that some vaccinations appear to be specifically associated with an increased incidence of loss of antibody titers after cancer chemotherapy [5, 15, 25]. Similar to our study, de la Fuente Garcia et al. found a higher percentage of seroprotected patients against measles virus post‐treatment in comparison to that protected against *Clostridium tetani* [26]. However, earlier studies have been extremely diverse with regard to findings of post‐treatment specific antibody titers [5, 6, 7, 10, 26, 27, 28, 29, 30]. Part of this heterogeneity of results may be explained by differing underlying diagnoses and treatments, rapidly evolving treatment protocols over time, different age‐groups, and varying underlying vaccination schedules. In our study, low case numbers limited practical conclusions with regard to comparatively high MMR seroprevalence. For the same reason, differences in treatment intensity groups could not be reliably compared.

Immunological effects of anti‐neoplastic therapy are diverse, and vaccine responses are not limited to the humoral component of the immune system [1, 10]. However, measurement of specific IgG titers is often employed as a surrogate to evaluate vaccination efficacy. Adequate function of B‐ cells is a prerequisite for production of immunoglobulins. Different studies have evaluated effects of cancer therapy on the B‐cell compartment with varying results [25, 31, 32, 33]. While in some studies B‐cell recovery appears to be a relatively rapid process, effects on memory B‐cells which are important for vaccine‐related antibody levels seem to be more prolonged [33]. In this study B‐cell (CD19+) numbers had recovered by the time of antibody assessment for the majority of patients.

The strength of this study is to provide real‐life data on vaccination‐related post‐treatment IgG titers from patients graded by treatment intensity as per ITR‐3 [16, 17] and treated by modern treatment protocols. A main limitation is a lack of baseline pre‐treatment specific IgG titer measurements, which may cause an overestimation of chemotherapy effects on antibody titers [28]. There is also a lack of evidence regarding specific levels of protective antibody titers [34] and methodological differences between studies make interpretation of results difficult [1]. In their cohort of 187 Finnish 3‐year‐old healthy children who had received 1 MMR vaccine at age 11–19 months, Kontio et al. described seropositivity of 100% to rubella virus, > 97% for measles virus, and 84% for mumps virus [35]. Persistence of MMR antibodies has been well documented [36, 37]. Immunogenicity and changes in specific antibody levels over time for the DTaP vaccine have previously been extensively described also in young children of Scandinavian origin [23, 24, 37, 38, 39, 40]. All children participating in this study were fully vaccinated with MMR and DTaP vaccines for their age range. In spite of treatment intensity grading as per ITR‐3, the heterogeneity of age, diagnoses and treatment regimens in this cohort of limited size must be borne in mind when interpreting the data.

Survivors of childhood cancer have been shown to experience excess mortality from infections even into adulthood [41]. Evidence‐based re‐vaccination practices are therefore essential in this vulnerable population. In view of our data, DTaP booster vaccinations after therapy are needed for a considerable proportion of patients. Since MMR is a live vaccine, there are concerns of administering it too soon after completion of cancer chemotherapy. In our study, 95% of cases showed positive antibody levels against measles and similar rates against mumps and rubella. Given the low number of cases in this study, there is a case for individualized measles, mumps and rubella re‐vaccination planning based on serology and risk‐profiling, even in an environment with extensive herd‐immunity. More research is warranted, especially in the form of sufficiently large prospective studies.

## Author Contributions

**Susanna Sundell:** investigation, methodology, formal analysis, writing original draft. **Miia Laine:** methodology, validation, investigation, writing–review and editing. **Liisa Järvelä:** conceptualization, methodology, writing–review and editing. **Ville Peltola:** conceptualization, methodology, writing–review and editing, supervision. **Tytti Vuorinen:** methodology, validation, investigation, writing–review and editing. **Päivi Lähteenmäki:** conceptualization, methodology, writing–review and editing, resources, project administration, supervision. **Linnea Schuez‐Havupalo:** conceptualization, methodology, formal analysis, writing of original draft and review/editing, project administration, supervision, funding acquisition.

## Consent

Permission to use the electronic patient record data obtained during standard clinical practice was obtained for this study from the hospital district of Southwest Finland, according to the institutional procedure (permit number TO8/027/19). According to the Finnish legislation, secondary use of patient record data in register studies does not require ethical board evaluation or informed consent.

## Conflicts of Interest

The authors declare no conflicts of interest.

## Supporting information


**Data S1.** Supporting Information.

## Data Availability

The data that support the findings of this study are available on request from the corresponding author. The data are not publicly available due to privacy or ethical restrictions.
