# Supplementary material for: Humoral Immunity to Measles, Mumps, Rubella, Diphtheria, Tetanus and Pertussis After Cancer Treatment in Children
Source: Cancer Rep (Hoboken). 2025 May 19;8(5):e70155. doi: 10.1002/cnr2.70155 (PMC12086975; doi:10.1002/cnr2.70155)
Supplement: Supplementary file 1 — Data S1. Supporting Information. [file CNR2-8-e70155-s001.docx]

TABLE S1 Diphtheria, tetanus, pertussis, measles, mumps, rubella vaccines in the Finnish national vaccination program

| Time point | Vaccine |
| --- | --- |
| 3,5, and 12 months | DTaP-IPV-Hib |
| 12-18 months | MMR |
| 4 years | DTaP-IPV |
| 6 years | MMR |
| 14-15 years | DTaP |

DTaP: diphtheria, tetanus, acellular pertussis

IPV: inactivated polio vaccine

Hib: *Hemophilus Influenzae B*

MMR: measles, mumps, rubella

| TABLE S2  A  Adjusted logistic regression: Risk of below-reference post-treatment tetanus antibody levels | | | |
| --- | --- | --- | --- |
| Variable | aOR | 95% CI | P-values |
| Treatment intensity group (high vs. low) | 0.53 | 0.12-2.36 | 0.41 |
| Total IgG (below vs. within reference) | 0.55 | 0.06-5.39 | 0.61 |
| Time since previous DTaP vaccine | 1.12 | 0.86-1.47 | 0.40 |
|  | | | |

| B  Adjusted logistic regression: Risk of below-reference post-treatment pertussis antibody levels | | | |
| --- | --- | --- | --- |
| Variable | aOR | 95% CI | P-values |
| Treatment intensity group (high vs. low) | 2.66 | 0.24-29.43 | 0.43 |
| Total IgG (below vs. within reference) | 0.57 | 0.03-9.81 | 0.70 |
| Time since previous DTaP vaccine | 1.36 | 0.94-1.96 | 0.11 |
|  | | | |

| C  Adjusted logistic regression: Risk of below-reference post-treatment diphtheria antibody levels | | | | | | | | | | |  |
| --- | --- | --- | --- | --- | --- | --- | --- | --- | --- | --- | --- |
| Variable | | aOR | | | 95% CI | | | P-values | | |  |
| Treatment intensity group (high vs. low) | | 0.62 | | | 0.14-2.76 | | | 0.53 | | |  |
| Total IgG (below vs. within reference) | | 0.72 | | | 0.09-5.66 | | | 0.75 | | |  |
| Time since previous DTaP vaccine | | 0.84 | | | 0.64-1.11 | | | 0.22 | | |  |
|  | | | | | | | | | | |  |
| D  Adjusted logistic regression: Risk of below-reference post-treatment mumps antibody levels | | | | | | | | | | |  |
| Variable | | | aOR | | 95% CI | | | P-values | | |  |
| Treatment intensity group (high vs. low) | | | 6.45 | | 0.53-78.21 | | | 0.14 | | |  |
| Time since previous MPR vaccine | | | 0.60 | | 0.27-1.35 | | | 0.22 | | |  |
| *Note.* 1–6-year-old children | | | | | | | | | | |  |
| E  Adjusted logistic regression: Risk of below-reference post-treatment rubella antibody levels | | | | | | | | | | | |
| Variable | | | aOR | | | | 95% CI | | | P-values | |
| Treatment intensity group (high vs. low) | | | 1.58 | | | | 0.21-12.05 | | | 0.66 | |
| Time since previous MPR vaccine | | | 0.82 | | | | 0.46-1.48 | | | 0.52 | |
| *Note.* 1–6-year-old children | | | | | | | | | | | |

TABLE S3 Time since previous DtaP/ MMR vaccination according to antibody status.

|  | N | Mean time from previous vaccine (years) | SD | p-value |
| --- | --- | --- | --- | --- |
| Tetanus antibodies within the reference range | 35 | 2.77 | 3.12 | 0.36 |
| Below-reference tetanus antibodies | 13 | 1.92 | 1.80 |  |
| Pertussis antibodies within the reference range | 5 | 4.20 | 2.86 | 0.07 |
| Below-reference pertussis antibodies | 31 | 2.10 | 2.27 |  |
| Diphtheria antibodies within the reference range | 17 | 2.18 | 1.94 | 0.21 |
| Below-reference diphtheria antibodies | 17 | 3.41 | 3.45 |  |
| Mumps antibodies within the reference range | 17 | 5.06 | 3.50 | NA |
| Below-reference mumps antibodies | 1 | 1.0 |  |  |
| Measles antibodies within the reference range | 18 | 5.11 | 3.56 | NA |
| Below-reference measles antibodies | 1 | 3.00 |  |  |
| Rubella antibodies within the reference range | 17 | 5.00 | 3.55 | NA |
| Below-reference rubella antibodies | 1 | 2.00 |  |  |

*Note:* Comparison of time since previous vaccination between cases with specific antibody levels within the reference range versus those with specific antibody levels below reference (T-test). Included are all cases who received a full vaccination series (at least 3 DTaP vaccines or 2 MMR vaccines)

NA: not applicable
